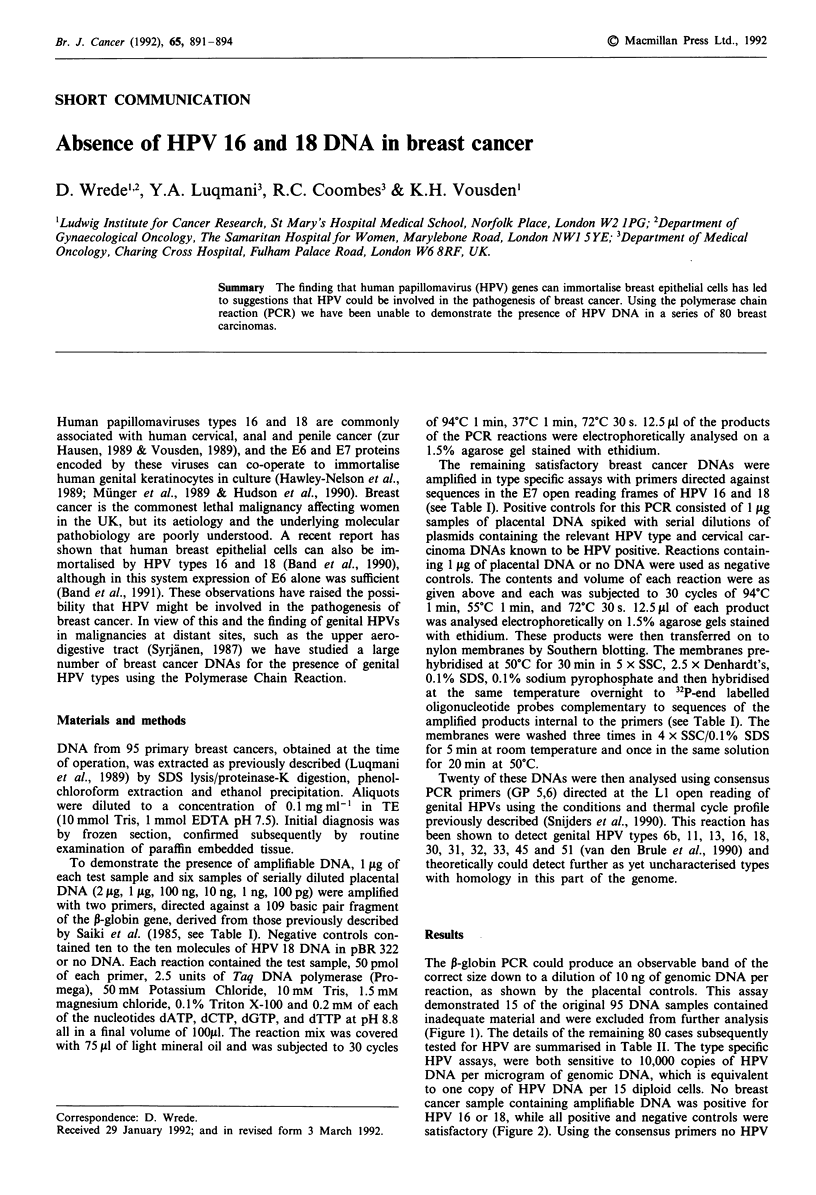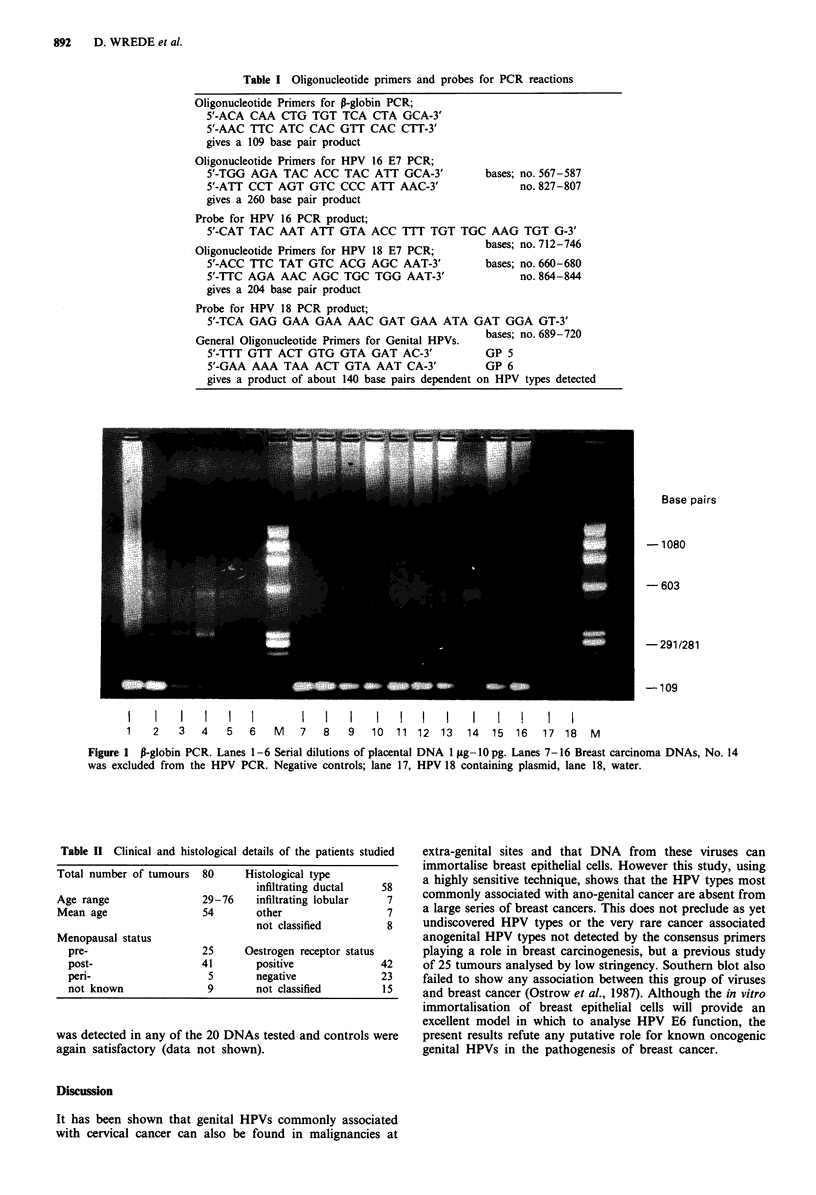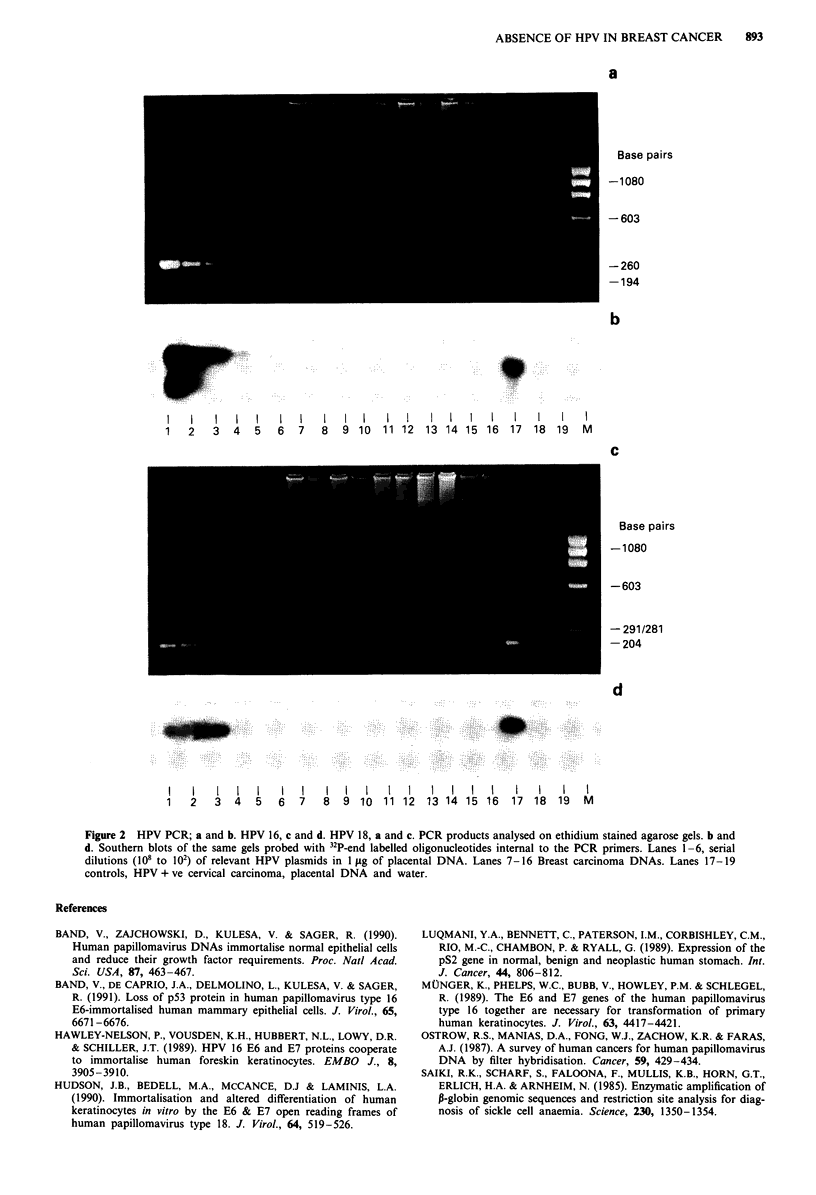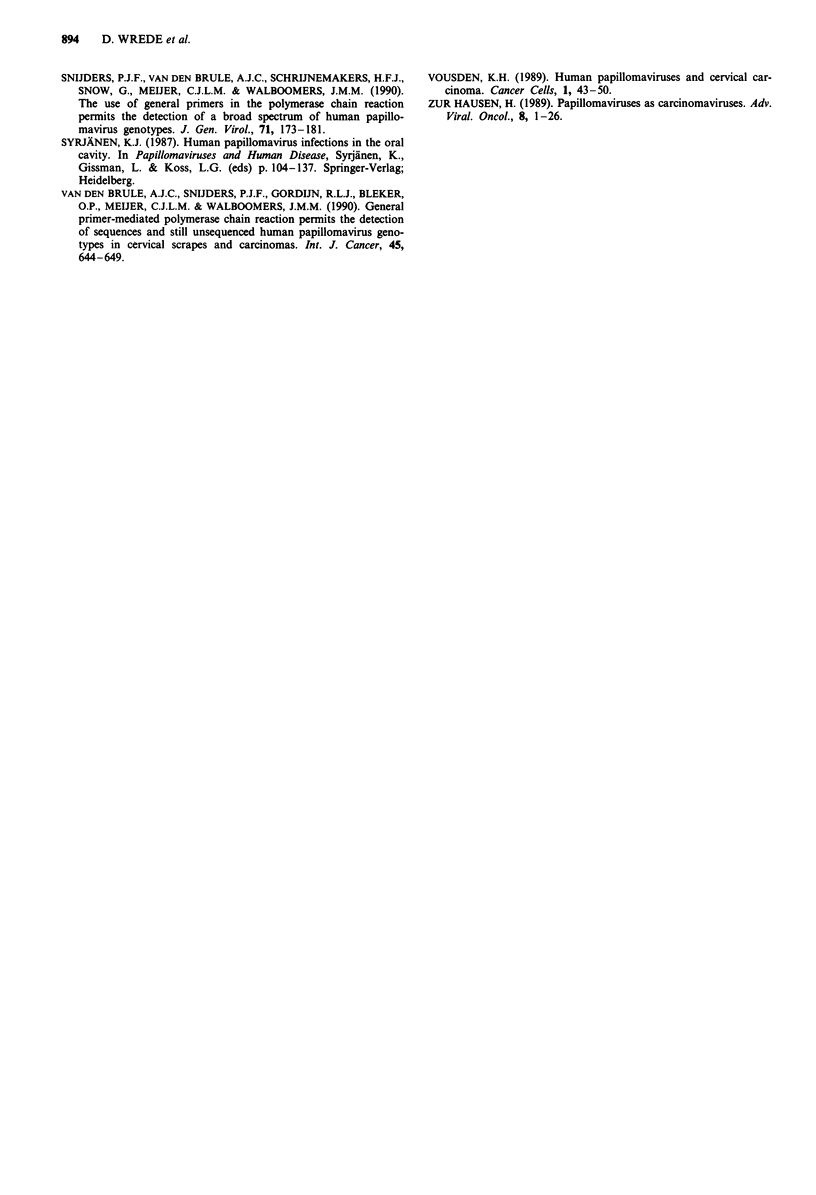# Absence of HPV 16 and 18 DNA in breast cancer.

**DOI:** 10.1038/bjc.1992.186

**Published:** 1992-06

**Authors:** D. Wrede, Y. A. Luqmani, R. C. Coombes, K. H. Vousden

**Affiliations:** Ludwig Institute for Cancer Research, St Mary's Hospital Medical School, London, UK.

## Abstract

**Images:**


Br. J. Cancer (1992), 65, 891-894 © Macmillan Press Ltd., 1992

SHORT COMMUNICATION

### Absence of HPV 16 and 18 DNA in breast cancer

D. Wrede[1,2], Y.A. Luqmani[3], R.C. Coombes[3] & K.H. Vousden[1]

[1]*Ludwig Institute for Cancer Research, St Mary's Hospital Medical School, Norfolk Place, London W2 1PG;* [2]*Department of Gynaecological Oncology, The Samaritan Hospital for Women, Marylebone Road, London NW1 5YE;* [3]*Department of Medical Oncology, Charing Cross Hospital, Fulham Palace Road, London W6 8RF, UK.*

**Summary** The finding that human papillomavirus (HPV) genes can immortalise breast epithelial cells has led to suggestions that HPV could be involved in the pathogenesis of breast cancer. Using the polymerase chain reaction (PCR) we have been unable to demonstrate the presence of HPV DNA in a series of 80 breast carcinomas.

Human papillomaviruses types 16 and 18 are commonly associated with human cervical, anal and penile cancer (zur Hausen, 1989 & Vousden, 1989), and the E6 and E7 proteins encoded by these viruses can co-operate to immortalise human genital keratinocytes in culture (Hawley-Nelson *et al.*, 1989; Münger *et al.*, 1989 & Hudson *et al.*, 1990). Breast cancer is the commonest lethal malignancy affecting women in the UK, but its aetiology and the underlying molecular pathobiology are poorly understood. A recent report has shown that human breast epithelial cells can also be immortalised by HPV types 16 and 18 (Band *et al.*, 1990), although in this system expression of E6 alone was sufficient (Band *et al.*, 1991). These observations have raised the possibility that HPV might be involved in the pathogenesis of breast cancer. In view of this and the finding of genital HPVs in malignancies at distant sites, such as the upper aerodigestive tract (Syrjänen, 1987) we have studied a large number of breast cancer DNAs for the presence of genital HPV types using the Polymerase Chain Reaction.

### Materials and methods

DNA from 95 primary breast cancers, obtained at the time of operation, was extracted as previously described (Luqmani *et al.*, 1989) by SDS lysis/proteinase-K digestion, phenol-chloroform extraction and ethanol precipitation. Aliquots were diluted to a concentration of 0.1 mg ml$^{-1}$ in TE (10 mmol Tris, 1 mmol EDTA pH 7.5). Initial diagnosis was by frozen section, confirmed subsequently by routine examination of paraffin embedded tissue.

To demonstrate the presence of amplifiable DNA, 1 μg of each test sample and six samples of serially diluted placental DNA (2 μg, 1 μg, 100 ng, 10 ng, 1 ng, 100 pg) were amplified with two primers, directed against a 109 basic pair fragment of the β-globin gene, derived from those previously described by Saiki *et al.* (1985, see Table I). Negative controls contained ten to the ten molecules of HPV 18 DNA in pBR 322 or no DNA. Each reaction contained the test sample, 50 pmol of each primer, 2.5 units of *Taq* DNA polymerase (Promega), 50 mM Potassium Chloride, 10 mM Tris, 1.5 mM magnesium chloride, 0.1% Triton X-100 and 0.2 mM of each of the nucleotides dATP, dCTP, dGTP, and dTTP at pH 8.8 all in a final volume of 100μl. The reaction mix was covered with 75 μl of light mineral oil and was subjected to 30 cycles of 94°C 1 min, 37°C 1 min, 72°C 30 s. 12.5 μl of the products of the PCR reactions were electrophoretically analysed on a 1.5% agarose gel stained with ethidium.

The remaining satisfactory breast cancer DNAs were amplified in type specific assays with primers directed against sequences in the E7 open reading frames of HPV 16 and 18 (see Table I). Positive controls for this PCR consisted of 1 μg samples of placental DNA spiked with serial dilutions of plasmids containing the relevant HPV type and cervical carcinoma DNAs known to be HPV positive. Reactions containing 1 μg of placental DNA or no DNA were used as negative controls. The contents and volume of each reaction were as given above and each was subjected to 30 cycles of 94°C 1 min, 55°C 1 min, and 72°C 30 s. 12.5 μl of each product was analysed electrophoretically on 1.5% agarose gels stained with ethidium. These products were then transferred on to nylon membranes by Southern blotting. The membranes prehybridised at 50°C for 30 min in 5 × SSC, 2.5 × Denhardt's, 0.1% SDS, 0.1% sodium pyrophosphate and then hybridised at the same temperature overnight to $^{32}$P-end labelled oligonucleotide probes complementary to sequences of the amplified products internal to the primers (see Table I). The membranes were washed three times in 4 × SSC/0.1% SDS for 5 min at room temperature and once in the same solution for 20 min at 50°C.

Twenty of these DNAs were then analysed using consensus PCR primers (GP 5,6) directed at the L1 open reading of genital HPVs using the conditions and thermal cycle profile previously described (Snijders *et al.*, 1990). This reaction has been shown to detect genital HPV types 6b, 11, 13, 16, 18, 30, 31, 32, 33, 45 and 51 (van den Brule *et al.*, 1990) and theoretically could detect further as yet uncharacterised types with homology in this part of the genome.

### Results

The β-globin PCR could produce an observable band of the correct size down to a dilution of 10 ng of genomic DNA per reaction, as shown by the placental controls. This assay demonstrated 15 of the original 95 DNA samples contained inadequate material and were excluded from further analysis (Figure 1). The details of the remaining 80 cases subsequently tested for HPV are summarised in Table II. The type specific HPV assays, were both sensitive to 10,000 copies of HPV DNA per microgram of genomic DNA, which is equivalent to one copy of HPV DNA per 15 diploid cells. No breast cancer sample containing amplifiable DNA was positive for HPV 16 or 18, while all positive and negative controls were satisfactory (Figure 2). Using the consensus primers no HPV

Correspondence: D. Wrede.
Received 29 January 1992; and in revised form 3 March 1992.

**Table I** Oligonucleotide primers and probes for PCR reactions

| | |
|---|---|
| Oligonucleotide Primers for β-globin PCR; | |
| 5'-ACA CAA CTG TGT TCA CTA GCA-3' | |
| 5'-AAC TTC ATC CAC GTT CAC CTT-3' | |
| gives a 109 base pair product | |
| Oligonucleotide Primers for HPV 16 E7 PCR; | |
| 5'-TGG AGA TAC ACC TAC ATT GCA-3' | bases; no. 567–587 |
| 5'-ATT CCT AGT GTC CCC ATT AAC-3' | no. 827–807 |
| gives a 260 base pair product | |
| Probe for HPV 16 PCR product; | |
| 5'-CAT TAC AAT ATT GTA ACC TTT TGT TGC AAG TGT G-3' | bases; no. 712–746 |
| Oligonucleotide Primers for HPV 18 E7 PCR; | |
| 5'-ACC TTC TAT GTC ACG AGC AAT-3' | bases; no. 660–680 |
| 5'-TTC AGA AAC AGC TGC TGG AAT-3' | no. 864–844 |
| gives a 204 base pair product | |
| Probe for HPV 18 PCR product; | |
| 5'-TCA GAG GAA GAA AAC GAT GAA ATA GAT GGA GT-3' | bases; no. 689–720 |
| General Oligonucleotide Primers for Genital HPVs. | |
| 5'-TTT GTT ACT GTG GTA GAT AC-3' | GP 5 |
| 5'-GAA AAA TAA ACT GTA AAT CA-3' | GP 6 |
| gives a product of about 140 base pairs dependent on HPV types detected | |

**Figure 1** β-globin PCR. Lanes 1–6 Serial dilutions of placental DNA 1 μg–10 pg. Lanes 7–16 Breast carcinoma DNAs, No. 14 was excluded from the HPV PCR. Negative controls; lane 17, HPV 18 containing plasmid, lane 18, water.

**Table II** Clinical and histological details of the patients studied

| | | | |
|---|---|---|---|
| Total number of tumours | 80 | Histological type | |
| | | infiltrating ductal | 58 |
| Age range | 29–76 | infiltrating lobular | 7 |
| Mean age | 54 | other | 7 |
| | | not classified | 8 |
| Menopausal status | | | |
| pre- | 25 | Oestrogen receptor status | |
| post- | 41 | positive | 42 |
| peri- | 5 | negative | 23 |
| not known | 9 | not classified | 15 |

was detected in any of the 20 DNAs tested and controls were again satisfactory (data not shown).

### Discussion

It has been shown that genital HPVs commonly associated with cervical cancer can also be found in malignancies at extra-genital sites and that DNA from these viruses can immortalise breast epithelial cells. However this study, using a highly sensitive technique, shows that the HPV types most commonly associated with ano-genital cancer are absent from a large series of breast cancers. This does not preclude as yet undiscovered HPV types or the very rare cancer associated anogenital HPV types not detected by the consensus primers playing a role in breast carcinogenesis, but a previous study of 25 tumours analysed by low stringency. Southern blot also failed to show any association between this group of viruses and breast cancer (Ostrow *et al.*, 1987). Although the *in vitro* immortalisation of breast epithelial cells will provide an excellent model in which to analyse HPV E6 function, the present results refute any putative role for known oncogenic genital HPVs in the pathogenesis of breast cancer.

**Figure 2** HPV PCR; **a** and **b**. HPV 16, **c** and **d**. HPV 18, **a** and **c**. PCR products analysed on ethidium stained agarose gels. **b** and **d**. Southern blots of the same gels probed with $^{32}$P-end labelled oligonucleotides internal to the PCR primers. Lanes 1–6, serial dilutions ($10^8$ to $10^2$) of relevant HPV plasmids in 1 μg of placental DNA. Lanes 7–16 Breast carcinoma DNAs. Lanes 17–19 controls, HPV + ve cervical carcinoma, placental DNA and water.

### References

BAND, V., ZAJCHOWSKI, D., KULESA, V. & SAGER, R. (1990). Human papillomavirus DNAs immortalise normal epithelial cells and reduce their growth factor requirements. *Proc. Natl Acad. Sci. USA*, **87**, 463–467.

BAND, V., DE CAPRIO, J.A., DELMOLINO, L., KULESA, V. & SAGER, R. (1991). Loss of p53 protein in human papillomavirus type 16 E6-immortalised human mammary epithelial cells. *J. Virol.*, **65**, 6671–6676.

HAWLEY-NELSON, P., VOUSDEN, K.H., HUBBERT, N.L., LOWY, D.R. & SCHILLER, J.T. (1989). HPV 16 E6 and E7 proteins cooperate to immortalise human foreskin keratinocytes. *EMBO J.*, **8**, 3905–3910.

HUDSON, J.B., BEDELL, M.A., MCCANCE, D.J & LAMINIS, L.A. (1990). Immortalisation and altered differentiation of human keratinocytes *in vitro* by the E6 & E7 open reading frames of human papillomavirus type 18. *J. Virol.*, **64**, 519–526.

LUQMANI, Y.A., BENNETT, C., PATERSON, I.M., CORBISHLEY, C.M., RIO, M.-C., CHAMBON, P. & RYALL, G. (1989). Expression of the pS2 gene in normal, benign and neoplastic human stomach. *Int. J. Cancer*, **44**, 806–812.

MÜNGER, K., PHELPS, W.C., BUBB, V., HOWLEY, P.M. & SCHLEGEL, R. (1989). The E6 and E7 genes of the human papillomavirus type 16 together are necessary for transformation of primary human keratinocytes. *J. Virol.*, **63**, 4417–4421.

OSTROW, R.S., MANIAS, D.A., FONG, W.J., ZACHOW, K.R. & FARAS, A.J. (1987). A survey of human cancers for human papillomavirus DNA by filter hybridisation. *Cancer*, **59**, 429–434.

SAIKI, R.K., SCHARF, S., FALOONA, F., MULLIS, K.B., HORN, G.T., ERLICH, H.A. & ARNHEIM, N. (1985). Enzymatic amplification of β-globin genomic sequences and restriction site analysis for diagnosis of sickle cell anaemia. *Science*, **230**, 1350–1354.

SNIJDERS, P.J.F., VAN DEN BRULE, A.J.C., SCHRIJNEMAKERS, H.F.J., SNOW, G., MEIJER, C.J.L.M. & WALBOOMERS, J.M.M. (1990). The use of general primers in the polymerase chain reaction permits the detection of a broad spectrum of human papillomavirus genotypes. *J. Gen. Virol.*, **71**, 173–181.

SYRJÄNEN, K.J. (1987). Human papillomavirus infections in the oral cavity. In *Papillomaviruses and Human Disease*, Syrjänen, K., Gissman, L. & Koss, L.G. (eds) p. 104–137. Springer-Verlag; Heidelberg.

VAN DEN BRULE, A.J.C., SNIJDERS, P.J.F., GORDIJN, R.L.J., BLEKER, O.P., MEIJER, C.J.L.M. & WALBOOMERS, J.M.M. (1990). General primer-mediated polymerase chain reaction permits the detection of sequences and still unsequenced human papillomavirus genotypes in cervical scrapes and carcinomas. *Int. J. Cancer*, **45**, 644–649.

VOUSDEN, K.H. (1989). Human papillomaviruses and cervical carcinoma. *Cancer Cells*, **1**, 43–50.

ZUR HAUSEN, H. (1989). Papillomaviruses as carcinomaviruses. *Adv. Viral. Oncol.*, **8**, 1–26.